# Outbreak of Multidrug-resistant *Pseudomonas Aeruginosa* Endophthalmitis Due to Contaminated Trypan Blue Solution

**DOI:** 10.18502/jovr.v14i3.4781

**Published:** 2019-07-18

**Authors:** Pritam Bawankar, Harsha Bhattacharjee, Manabjyoti Barman, Ronel Soibam, Hemalata Deka, Ganesh Chandra Kuri, Jnanankar Medhi

**Affiliations:** ^1^Department of Vitreo-Retina Surgery, Sri Sankaradeva Nethralaya, Guwahati, Assam, India; ^2^Department of Oculoplastic, Sri Sankaradeva Nethralaya, Guwahati, Assam, India; ^3^Department of Cornea, Sri Sankaradeva Nethralaya, Guwahati, Assam, India

**Keywords:** Multidrug Resistance, Pars Plana Vitrectomy, Post-cataract Surgery Endophthalmitis, Pseudomonas Aeruginosa, Small-incision Cataract Surgery

## Abstract

**Purpose:**

To report the investigation of an outbreak of multidrug-resistant (MDR) *Pseudomonas aeruginosa *endophthalmitis in 13 patients after cataract surgery and to emphasize on the importance of clinical profile, risk factors, and treatment outcomes

**Methods:**

This was a hospital-based, retrospective case study with 13 consecutive patients who had man- ual small-incision cataract surgery with intraocular lens (IOL) implantation and developed acute postoperative *Pseudomonas aeruginosa* endophthalmitis. The anterior chamber taps, vitreous aspirates, and environ- mental surveillance specimens were inoculated for culturing. Antibiotic susceptibility testing was performed using the agar diffusion method. Pulsed-field gel electrophoresis (PFGE) was used to determine the relation- ship between bacterial isolates recovered from study patients and contaminated surveillance samples.

**Results:**

*Pseudomonas aeruginosa *was isolated from all 13 eyes with acute postoperative endophthalmitis and the trypan blue solutions used during surgery. Sensitivity tests revealed that all isolates had an identical resistance to multiple drugs and were only susceptible to imipenem. Genomic DNA typing of *Pseudomonas aeruginosa *isolates recovered from patients and trypan blue solutions showed an identical banding pattern on the PFGE. Despite the prompt use of intravitreal antibiotics and early vitrectomy with IOL explantation in some patients, the outcome was poor in about 50% of patients.

**Conclusion:**

Positive microbiology and genomic DNA typing results proved that the contaminated trypan blue solutions were the source of infection in this outbreak. Postoperative endophthalmitis caused by *Pseudomonas aeruginosa *is often associated with a poor visual prognosis despite prompt treatment with intravitreal antibiotics.

##  INTRODUCTION

Approximately 12.5 million people are blind in India and cataract is the main contributor to this striking number accounting for 50–80% of cases.^[1]^ Manual small-incision cataract surgery (MSICS) is a faster, less expensive small-incision form of extracapsular cataract extraction (ECCE) that is principally used in the developing world.^[2]^ MSICS has the advantage of a self-sealing sutureless wound and is less dependent on expensive technology than phacoemulsification. With the improvement in preoperative prophylactic measures, sterilization protocols, surgical techniques, and good postoperative care, the infection rate after cataract surgery has decreased. However, there is still the possibility of infections, and the most devastating is endophthalmitis.

Postoperative endophthalmitis is a catastrophic complication of intraocular surgery mainly associated with cataract extraction and intraocular lens (IOL) implantation, with an incidence of 0.08 to 1%.^[3]^ The most common causative organisms are gram-positive coagulase negative bacteria; however, gram-negative organisms have been isolated from 6% to 29% of cases in larger reported series.^[4]^ Despite the low prevalence of gram-negative organisms, a more vigorous management approach including early vitrectomy is required, as these organisms are highly virulent.

*Pseudomonas aeruginosa *is a gram-negative bacillus, commonly found in soil and moist environments, which has emerged as an important opportunistic pathogen in hospitalized and immunocompromised patients affecting many anatomic sites, such as the skin, ears, lungs, heart, urinary tract, bone, and eyes.^[5]^The most common ocular infection caused by *Pseudomonas aeruginosa *is contact lens-associated keratitis, but it may also cause post-surgical acute endophthalmitis.^[6]^ Outbreaks of acute postoperative *Pseudomonas aeruginosa *endophthalmitis have been described in numerous case studies, most of which were caused by bacteria from air, IOLs, inadequately sterilized irrigation fluid, and surgical equipment.^[7,8]^

Trypan blue is used for staining the anterior capsule during cataract surgery. Eyes with mature cataracts and conditions resulting in a compromised red reflex such as corneal scarring and edema, asteroid hyalosis, vitreous hemorrhage, and retinal disease have poor visualization of the anterior capsule during cataract surgery, making capsulorrhexis extremely difficult.^[9]^ Therefore, capsular staining with trypan blue is helpful in enhancing visualization in such eyes. However, the risk of bacterial infection after instilling trypan blue into the eye remains real.^[10]^

The purpose of this study was to investigate an outbreak of multidrug-resistant (MDR) *Pseudomonas aeruginosa *endophthalmitis after cataract surgery in 13 patients and determine the importance of associated risk factors, clinical profile, microbiological analysis, early diagnosis, and therapy.

##  METHODS

This study is a hospital-based, retrospective case study, approved by the local Institutional Review Board and conducted in accordance with the principles of the Helsinki Declaration. In March 2017, 13 patients (3 women and 10 men) who underwent standard MSICS with IOL implantation developed acute postoperative endophthalmitis and were referred to our tertiary eye center for further management. All 13 patients were operated in the same operation theater by five different experienced surgeons over a period of five days at a district hospital in north-east India. The medical records of all 13 patients treated for multidrug-resistant *Pseudomonas aeruginosa *endophthalmitis after cataract surgery were reviewed.

The following data were extracted from the patients' medical records for this study: demographic information, eye affected, best corrected visual acuity (BCVA) at the time of diagnosis of endophthalmitis, date and type of anesthesia and surgery, type of IOL implanted, time between cataract surgery and diagnosis of endophthalmitis, presenting signs and symptoms, site of culture, antibiotic sensitivity testing, treatment, and outcomes.

A 0.1 mL anterior chamber sample was collected from 10 patients using a 30-gauge needle and syringe before the administration of intravitreal antibiotics, while vitreous samples were collected from three patients at the time of pars plana vitrectomy and sent to the microbiology laboratory. Cultures and smears for the detection of bacterial and fungal agents were carried out on the intraocular specimens within 30 minutes of their collection. The culture media used were blood agar (5% sheep), chocolate agar (5% sheep blood), brain heart infusion broth, thioglycollate broth, and Sabouraud dextrose agar (Hi-Media, Mumbai). All the inoculated media were incubated at 37ºC except for Sabouraud dextrose agar, which was incubated at 25ºC. Chocolate agar was incubated in an atmosphere of 10% CO${}_{2}$ (anaerobic system Mark V Jar, Hi-Media). Gram and Giemsa stains for cytology and potassium hydroxide (KOH) preparation for detecting fungi were prepared from the anterior chamber tap and vitreous specimens.

The criteria used to identify the isolated organism as the causative agent were: (i) growth on a single medium correlating with direct smear findings, (ii) growth of the same organism on two or more of the inoculated media, or (iii) confluent growth in any solid medium, or a combination of these criteria. Antibiotic susceptibility to cephotaxime, cefuroxime, ceftriaxone, ceftazi- dime, chloramphenicol, vancomycin, amikacin, tobramycin, ciprofloxacin, moxifloxacin, co-trimoxazole, imipenem, and piperacillin-tazobac- tam was tested using the classic agar diffusion (Kirby–Bauer) method. Furthermore, during the outbreak, microbiological analysis was also performed on the surveillance samples from the unopened bottles of povidone–iodine solution (Apidine-5 Appasamy Associates, Arumbakkam, Chennai, India), trypan blue solution (Sunblue, Unison Pharmaceuticals, Ahmedabad, Gujarat, India), hydroxypropyl methylcellulose ophthalmic solution (Eyevisc, Bio-Tech Ophthalmicus Pvt. Ltd., Gandhinagar, Gujrat, India), and the irrigation solutions [Ringer's lactate (RL, Schwitz Biotech, Ahmedabad, Gujarat, India)] from the same batch as used for the surgery.

In addition, the polymethylmethacrylate (PMMA, Biovision, Biotech Vision Care Pvt. Ltd. Ahmedabad, Gujarat, India) intraocular lenses, surgical instruments, dressings, air-conditioning system of the operating rooms, and autoclave efficacy were also tested. All the surveillance samples were obtained from the same operating theater during the endophthalmitis outbreak and were microbiologically analyzed using the protocol described by our laboratory in the hospital. Subsequently, smear and cultures of the patients' isolates, surveillance specimens, and bacterial isolates were genotyped to identify the similar isolates. To determine the relatedness of bacterial isolates, we analyzed the chromosomal restriction fragment patterns using pulsed-field gel electrophoresis (PFGE) with standard techniques.

All 13 patients were managed using the standard institutional protocol for the management of acute postoperative endophthalmitis. This essentially consisted of aqueous or vitreous sampling or both, microscopy, and culture sensitivity analysis of undiluted aqueous or vitreous samples, intravitreal antibiotics (vancomycin [1 mg/0.1 mL] plus ceftazidime [2.25 mg/0.1 mL]/N-formimidoyl-thienamycin (imipenem, 100 μg/0.1mL]), and vitrectomy. Intensive topical antibiotics (moxifloxacin 0.5% and tobramycin 0.3%) and a corticosteroid (prednisolone acetate 1%) were administered to all patients. Initial treatment included intravitreal antibiotic injection in seven eyes, pars plana vitrectomy with IOL explantation and injection of intravitreal antibiotics in four eyes, and evisceration in two eyes. Intravitreal imipenem was administered to five patients following culture and sensitivity reports. Additional procedures such as repeat intravitreal antibiotics or pars plana vitrectomy, corneal patch grafting, and tunnel wound repair were performed by the individual-treating physicians without a predefined study protocol.

##  RESULTS

Thirteen patients (10 men and 3 women) underwent MSICS with posterior chamber IOL implantation from March 7 to 11, 2017 at a district hospital in Northeast India. All 13 patients developed acute *Pseudomonas aeruginosa * endophthalmitis. The average age was 67 (range, 50–85) years. The right and left eyes were affected in seven and six patients, respectively. The mean interval between surgery and diagnosis of endophthalmitis was five (range, three–eight) days. The demographics, clinical settings, and culture sites are shown in Table 1. All 13 patients developed varying degrees of ocular discomfort and decreased vision from postoperative day 1 to 3. BCVA documented in 11 patients was perception of light and that of two patients was hand motion at the time of presentation. All patients had severe anterior chamber reaction and hypopyon at the time of presentation.

**Table:** Demographics, clinical findings and treatment outcomes in 13 patients with multidrug-resistant pseudomonas aeruginosa endophthalmitis after cataract surgery

**Patient No.**	**Age (yrs)/ Gender **	**Eye **	**Systemic Disease**	**Visual Acuity at the time of presentation **	**Days between Surgery and Endophthalmitis Diagnosis (Days)**	**Clinical Signs**	**Culture Site**	Treatment*	**Outcome**
1	60/F	OS	DM	LP	3	Eyelid edema, discharge, conjunctival congestion, chemosis, corneal edema, fibrinous exudates covering pupil, 2-mm hypopyon, tunnel infiltrate, exposed tunnel, ragged wound margin with iris tissue prolapsed	Vitreous	Day 1 – IV (V + CA) Day 2 – PPV + IOL Removal + IV (V + CA) Day 8 – IV (V + CA)	Phthisis bulbi after one month
2	76/M	OD	DM, HTN	LP	6	Eyelid edema, discharge, conjunctival congestion, chemosis, corneal haze with dense infiltrate, fibrinous exudates in AC, 3-mm hypopyon, tunnel infiltrate, necrosed tunnel, wound gaping with iris tissue prolapsed, IOP markedly raised	Aqueous	Day 1 – IV (V + CA)	No improvement, severe ocular pain, no LP; evisceration on day 2
3	55/M	OS	NIL	LP	3	Eyelid edema, discharge, conjunctival congestion, chemosis, corneal edema, 1-mm hypopyon	Vitreous, Aqueous	Day 1 – IV (V + CA) Day 2 – PPV + IOL Removal + IV (V + CA)	BCVA 3/60, AC quiet, VC 3+, second order vessel visible on fundoscopy after one month of PPV
4	75/M	OD	NIL	LP	7	Eyelid edema, discharge, conjunctival congestion, chemosis, corneal dense corneal infiltrate, fibrinous exudates covering pupil, 3-mm hypopyon, tunnel infiltrate, necrosed tunnel, wound gaping with iris tissue prolapsed, IOP markedly raised	Aqueous	Day 1 – IV (IM)	No improvement, severe ocular pain, no LP; evisceration on day 3
5	50/F	OD	NIL	LP	6	Discharge, conjunctival congestion, corneal edema, 2-mm hypopyon, membranous exudates on IOL	Aqueous, Vitreous	Day 1 – IV (V + CA) Day 2 – PPV + IOL Removal + FGE + SOI + IV (V + CA)	BCVA 6/60, AC quiet, silicon filled eye, retina healthy on fundoscopy after one month of PPV
6	50/F	OD	HTN	LP	5	Eyelid edema, discharge, conjunctival congestion, chemosis, corneal edema, severe fibrinous exudates in AC, 2-mm hypopyon	Vitreous	Day 1 – PPV + IOL Removal + FGE + LPFC + EL + SOI + IV (VA + CA) 18/04/17 – SOR + RR + MP + FGE + EL + RE SOI	BCVA 2/60, AC quiet, silicon filled eye, retina healthy on fundoscopy after three weeks of second surgery
7	75/M	OD	NIL	LP	5	Eyelid edema, discharge, conjunctival congestion, chemosis, dense corneal infiltrate, corneal edema with raised IOP, full chamber exudate, necrosed tunnel, wound gaping with incarcerated and exposed uveal tissue	Aqueous	Severe ocular pain, no LP; evisceration on Day 2
8	78/M	OS	HTN	LP	6	Discharge, conjunctival congestion, 1-mm hypopyon, fibrinous exudates on IOL	Aqueous, Vitreous	Day 1 – PPV + IOL removal + IV (V + CA)	BCVA 6/36, AC quiet, VC 2+, normal retinal findings on fundoscopy after one month of PPV
9	58/M	OD	NIL	LP	4	Eyelid edema, conjunctival congestion, chemosis, corneal edema, exudative membrane over pupil, 2-mm hypopyon	Vitreous	Day 1 – PPV + IOL Removal + IV (V + CA) Day 3 – IV (IM) Day 5 – IV (IM)	Retinal and choroidal detachment with proliferative vitreoretinopathy after three weeks; no further follow-up
10	85/M	OS	HTN, HEMIPARESIS	LP	6	Eyelid edema, discharge, conjunctival congestion, chemosis, dense corneal infiltrate, severe fibrinous exudates in AC, 3-mm hypopyon, tunnel infiltrate, necrosed tunnel with ragged edges, IOP markedly raised	Aqueous	Day 1 – IV (V + CA)	Severe ocular pain, no LP, restricted eye movement, high IOP; evisceration on day 2
11	75/M	OD	TB, RHD, MVP, HTN,	LP	8	Eyelid edema, discharge, conjunctival congestion, chemosis, corneal infiltrate, corneal edema, fibrinous exudates in AC, 3-mm hypopyon, tunnel infiltrate, exposed tunnel	Aqueous, Vitreous	Day 2 – PPV + IOL Removal + IV (IM)	No improvement, no LP; phthisis bulbi after six weeks
12	65/M	OS	NIL	HM	6	Eyelid edema, discharge, conjunctival congestion, chemosis, dense corneal infiltrate on nasal side of cornea, fibrinous exudates in AC covering pupil, 2-mm hypopyon	Aqueous	Day 1 – IV (V + CA) Day 3 – IV (IM) Day 5 – IV (IM) Day 7 – Corneal patch graft	BCVA 4/60, graft hazy, rest cornea clear, AC quiet, VC 3+, retina healthy after one month.
13	70/M	OS	NIL	HM	5	Discharge, conjunctival congestion, chemosis, corneal edema, exudates in AC, 1-mm hypopyon, wound gaping	Aqueous	Day 1 – IV (IM) Day 3 – IV (IM) 9/4/17 – IV (IM) 10/4/17 (gaping) Wound repair + IV (IM)	VA 6/60, cornea clear, AC quiet, VC 2+, retina healthy after one month
*Treatment started from the day of presentation and diagnosis (e.g., Day 1 – treatment received by the patient on the first day of presentation and diagnosis of endophthalmitis)									
AC, anterior chamber; BCVA, best corrected visual acuity; CA, ceftazidime; DM, diabetes mellitus; EL, endolaser; F, female; FGE, fluid gas exchange; HM, hand motion; HTN, hypertension; IM, imipenem; IOP, intraocular pressure; IV, intravitreal; LP, light perception; LPFC, liquid perflurocarban; M, male; MP, membrane peeling; MVP, mitral valve prolapse; OD, oculus dexter; OS, oculus sinister; PPV, pars plana vitrectomy; RHD, rheumatic heart disease; RR, retinal retinectomy; RE SOI, re-injection of silicone oil; SIO, silicone oil; SOR, silicone oil removal; TB, Tuberculosis; V, vancomycin; VC, vitreous cells									

Six eyes had exposed scleral-corneal tunnels with infiltrate, whereas eleven had corneal edema with raised intraocular pressure, and one patient developed dense corneal infiltrate over the nasal cornea. All patients had clinically dense vitreous haze and, thus, dark fundus reflex. Despite the prompt use of intravitreal antibiotics and early vitrectomy with IOL explantation, the outcome was poor in about 50% of the cases (evisceration and phthisis of four and two eyes, respectively, and retinal and choroidal detachment with extensive proliferative vitreoretinopathy in one eye). A moderate degree of improvement was observed in six eyes in the last control visit. Seven eyes underwent vitrectomy and IOL explantation with intravitreal antibiotics, including two cases that developed phthisis bulbi over a period of one month (cases 1 and 11), four cases that showed moderate improvement with a BCVA ranging from 2/60 to 6/36 (cases 3, 5, 6, and 8), and a case that developed retinal detachment with gross proliferative vitreoretinopathy did not come for follow-up after three weeks (case 9). Four eyes underwent evisceration.

**Figure 1:**
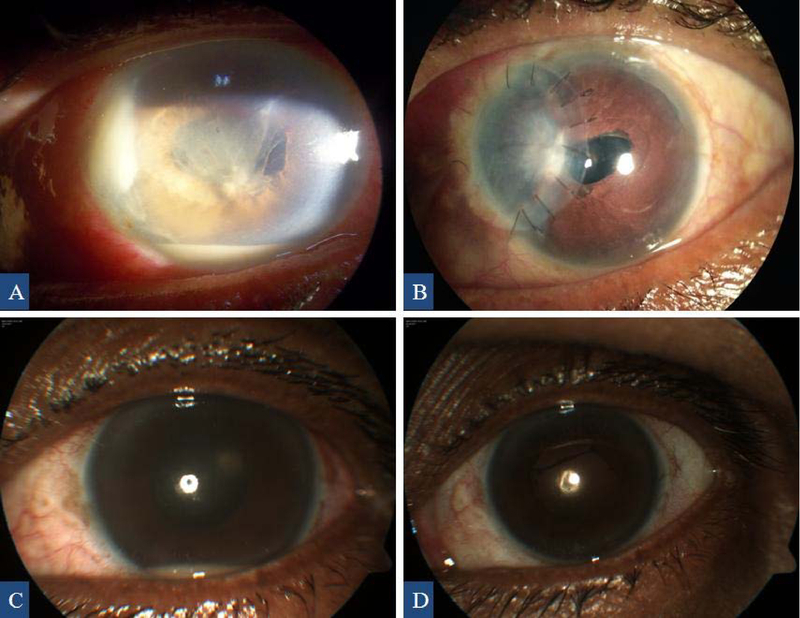
Anterior segment images showing nasal corneal abscess, hypopyon, and fibrinous exudates at the time of presentation (A) and corneal patch graft at the last visit (B) of case 12. Anterior segment images of case 13 showing recurrence of hypopyon (C) and postoperative image at the last visit with no hypopyon (D).

The remaining two eyes (cases 12 and 13) were treated with intravitreal* N*-formimidoyl-thienamycin (imipenem) injection, as the BCVA at the time of presentation was hand motion with a less severe anterior chamber reaction, and they showed moderate vision improvement. With these two eyes, case 12 had corneal patch graft for dense nasal corneal infiltrate [Figures 1(A) and (B)] and case 13 required suturing for exposed and gaped tunnel. In case 13, the resolution of hypopyon and good fundal reflex with a hazy view of disc was noted after two intravitreal injections of imipenem. After three weeks, the same patient (case 13) developed tunnel gaping, raised IOP, recurrence of hypopyon, and a dark fundal reflex. The patient underwent wound repair and intravitreal injection of imipenem and showed significant improvement in visual acuity and clinical picture during the following weeks [Figures 1(C) and (D)]. A summary of the clinical findings, treatment, and outcomes are presented in Table 1.

Anterior chamber taps were performed in all patients; whereas adequate samples could not be collected from three patients because of thick fibrinous exudates in the anterior chamber. Vitreous aspirate was collected from all patients who underwent vitrectomy and sent for culture. The anterior chamber and vitreous aspirates of all 13 patients yielded *Pseudomonas aeruginosa*, which was also isolated from the surveillance samples from the unopened bottles of trypan blue solution from the same batch as used for the surgery. On the other hand, no microbial contamination was found in samples of povidone–iodine solution, hydroxypropyl methylcellulose ophthalmic solution, PMMA IOLs, surgical instruments, dressings, air-conditioning system of the operating room, and the irrigation solution (Ringer's lactate).

Sensitivity tests revealed that all *Pseudomonas aeruginosa *eye isolates had an identical multidrug-resistance (MDR) susceptibility profile and were susceptible to imipenem but resistant to cephotaxime, cefuroxime, ceftriaxone, ceftazidime, chloramphenicol, vancomycin, amikacin, tobramycin, ciprofloxacin, moxifloxacin, co-trimoxazole, and piperacillin-tazobactam. Genomic DNA typing of *Pseudomonas aeruginosa* isolates recovered from the study patients and trypan blue solutions showed an identical banding pattern on ethidium bromide-stained gels. No other strains of *Pseudomonas aeruginosa *unrelated to the outbreak were typed on the PFGE.

##  DISCUSSION

The current study describes an outbreak of cataract surgery-related MDR *Pseudomonas aeruginosa* endophthalmitis caused by con- taminated trypan blue ophthalmic solution. The outcome was evisceration or phthisis in six (46%) of the thirteen eyes, and a moderate degree of improvement in six eyes in the last control visit. One patient who did not follow-up after three weeks was diagnosed with retinal and choroidal detachment with extensive proliferative vitreoretinopathy. *Pseudomonas aeruginosa* postoperative endophthalmitis is often associated with a poor visual prognosis even with early treatment with intravitreal antibiotics to which the isolates are susceptible.

Pinna et al^[11]^ reported an outbreak of post-cataract surgery endophthalmitis caused by *Pseudomonas aeruginosa *in 20 patients, and although intravitreal antibiotics were promptly administered, 10 patients had evisceration or phthisis of the affected eye, 5 eyes showed a minimal degree of improvement, and 7 patients did not follow-up within one week after intravitreal antibiotic injection. Zaluski et al^[12]^ reported four cases of *Pseudomonas aeruginosa *endophthalmitis caused by the contamination of the internal pathways of a phacoemulsifier; three of these four patients had evisceration or phthisis of the affected eye and one had visual acuity of 20/400. In a recent study by Eifrig et al,^[13]^ 18 of 28 eyes with *Pseudomonas aeruginosa *endophthalmitis either developed phthisis bulbi or were eviscerated, and none of the remaining nine patients achieved a final visual acuity of 5/200 or better.

*Pseudomonas aeruginosa* is one of the most common gram-negative pathogens associated with hospital-acquired infections, and the clinical evidence indicates that multidrug resistance of this organism is growing and affecting the selection of proper treatment.^[14]^ The increasing antibiotic resistance of *Pseudomonas aeruginosa* involves several mechanisms including efflux pump over expression, decreased outer membrane permeability, production of metallo-beta-lactamases, and structural alterations of topoisomerases II and IV, which are involved in quinolone resistance.^[15]^ The current study confirmed that the detection of ocular *Pseudomonas aeruginosa* isolate with multiple antibiotic resistance is common. In fact, multidrug resistance to cefazolin, chloramphenicol, tetracycline, aminoglycosides, piperacillin-tazobactam, and fluoroquinolones was observed with all isolates. This occurrence may, in part, explain the poor outcome in our patients, despite prompt treatment with intravitreal antibiotics.

Imipenem is a member of the beta-lactam class of antibiotics, which kills bacteria by binding to penicillin-binding proteins and inhibiting cell wall synthesis, exhibiting a broader spectrum of activity than that of cephalosporins and penicillins.^[16]^ Imipenem is regarded as an agent of choice in the treatment of severe nosocomial infections caused by sensitive strains of *Pseudomonas aeruginosa*.^[16]^ In the current study,* Pseudomonas aeruginosa * strains isolated from patients showed multidrug resistance except against imipenem.

In our study, despite the prompt use of intravitreal antibiotics, the outcome was poor in about 50% of patients [evisceration (cases 2, 4, 7, and 10) and phthisis (1 and 11)]. In all these cases, the sclerocorneal tunnel showed ragged wound margin, tunnel infiltrate, gaping with iris tissue prolapse, and severe fibrinous exudates in the anterior chamber with 2–3 mm hypopyon at the time of presentation. Although the organisms isolated in all cases of endophthalmitis were the same, eyes with exposed and gaped tunnels presented with more fulminant manifestations and were ultimately blind. Poorly constructed and distorted wounds could enhance the chances of postsurgical anterior chamber contamination and play pivotal roles in increased rates of endophthalmitis.

Although the poorly constructed wounds did not cause the development of endophthalmitis in the current series, we believe that they were associated with additional ocular complications after the development of endophthalmitis, worsening the outcome. Several studies have reported increased rates of postsurgical endophthalmitis among male patients,^[17,18]^ those with low socioeconomic and immune-compromised status,^[19]^ older age (mean age of 81 years),^[20]^ diabetes mellitus,^[20]^ and in patients with postoperative wound defects.^[21,22]^ All these factors were present in the six patients who exhibited very poor outcome: two were diabetic (cases 1 and 2), one had a history of stroke (patient 10), and one had a history of tuberculosis, rheumatic heart disease, and mitral valve prolapse (patient 11). We believe that these factors may not only have predisposed the patients to the development of endophthalmitis but could have also caused a more fulminant course, which might be a possible explanation for the fulminant course in these patients.

*Pseudomonas aeruginosa *is not a normal commensal on the periocular skin or conjunctiva, and most associated epidemics reported in the literature seem to have an exogenous origin.^[11]^ *Pseudomonas aeruginosa* post cataract surgery endophthalmitis outbreaks have been reported in association with contaminated ophthalmic solutions used during the surgery (balanced salt solution, trypan blue, and hyaluronic acid),^[8,23,24,25]^ contaminated phacoprobe,^[21]^ and contamination of internal fluid pathways of a phacoemulsifier.^[12,26,27,28]^ In our survey, the analysis of the surveillance samples showed* Pseudomonas aeruginosa *contamination of trypan blue solution of the same batch used for the surgeries. On the other hand, no microbial contamination was found in other surveillance samples. Furthermore, we confirmed that the *Pseudomonas aeruginosa *isolated recovered from the study patients typed using PFGE were similar to the strains recovered from the trypan blue solution. Therefore, we believe that trypan blue solution is the culprit in causing the outbreak described.

There are some limitations to this study. The case data provided by the district hospital had no records of intraoperative complications such as posterior capsular rupture with vitreous loss, subconjunctival and intracameral antibiotics at the end of surgery, and the use of topical povidone–iodine in all cases of cataract surgery. Therefore, these factors could not be assessed in the current study. Nonetheless, even with these limitations, the analysis provides clear evidence to support the notion that MDR *Pseudomonas aeruginosa-*associated endophthalmitis is responsible for poor visual outcomes despite early and appropriate treatment. This occurrence of cluster endophthalmitis may be explained by the inoculation of a large bacterial burden and virulence of the organism. This case study provides additional information for primary care physicians, general ophthalmologists, and other eye care professionals regarding clinical profile, risk factors, early diagnosis, initiation of appropriate treatment and visual outcomes in cases of MDR postsurgical *Pseudomonas aeruginosa *endophthalmitis.

In conclusion, our case study confirmed that the outcome of cataract surgery-related endophthalmitis caused by MDR *Pseudomonas aeruginosa *was poor, despite prompt treatment with intravitreal broad-spectrum antibiotics. Susceptibility to imipenem suggests that this antibiotic may be a potential candidate for the treatment of ocular infections caused by multidrug-resistant *Pseudomonas aeruginosa*. Ophthalmologists should never assume that solutions or medications used intraoperatively are sterile and they should never be used for multiple operations. We also recommend the use of molecular biology techniques, such as PFGE, to confirm the source of infection.

##  Financial Support and Sponsorship

The authors would like to thank the Sri Kanchi Sankara Health and Educational Foundation for their support in completing the study.

##  Conflicts of Interest

There are no conflicts of interest.

## References

[1] Venkatesh R., Das M., Prashanth S., Muralikrishnan R. (2005). Manual small incision cataract surgery in eyes with white cataracts. *Indian Journal of Ophthalmology*.

[2] Kongsap P. (2011). Visual outcome of manual small-incision cataract surgery: comparison of modified Blumenthal and Ruit techniques. *Int J Ophthalmol*.

[3] Hanscom T. A. (2004). Postoperative Endophthalmitis. *Clinical Infectious Diseases*.

[4] Eifrig C. W. G., Scott I. U., Flynn H. W., Miller D. (2003). Endophthalmitis caused by *Pseudomonas aeruginosa*. *Ophthalmology*.

[5] Ohl CA., Pollack M., Kasper DL., Braunwald E., Fauci AS. (2005). Infections due to Pseudomonas species and related organisms. *Harrison's principles of internal medicine*.

[6] O'Brien TP., Hazlett LD., Pepose JS., Holland GN., Wilhelmus KR. (1996). Pathogenesis of ocular infection. *Ocular infection and immunity. St*.

[7] Taban M. (2005). Acute Endophthalmitis Following Cataract Surgery. *JAMA Ophtalmology*.

[8] Swaddiwudhipong W., Linlawan P., Prasantong R., Kiphati R., Wongwatcharapaiboon P. (2000). A report of an outbreak of postoperative endophthalmitis. *J Med Assoc Thai*.

[9] Chang DF. (2005). *Trypan blue versus indocyanine green: a clinical comparison of these dyes for capsular staining. Cataract Refract Surg Today*.

[10] Sunenshine R., Schultz M., Lawrence M., Shin S., Jensen B., Zubairi S., Labriola A., Shams A., Noble‐Wang J., Arduino M., Gordin F., Srinivasan A. (2009). An Outbreak of Postoperative Gram‐Negative Bacterial Endophthalmitis Associated with Contaminated Trypan Blue Ophthalmic Solution. *Clinical Infectious Diseases*.

[11] Pinna A., Usai D., Sechi L. A., Zanetti S., Jesudasan N. C., Thomas P. A., Kaliamurthy J. (2009). An Outbreak of Post-Cataract Surgery Endophthalmitis Caused by Pseudomonas aeruginosa. *Ophthalmology*.

[12] Zaluski S. (1999). Pseudomonas aeruginosa endophthalmitis caused by contamination of the internal fluid pathways of a phacoemulsifier. *Journal of Cataract & Refractive Surgery*.

[13] Eifrig C. W. G., Scott I. U., Flynn H. W., Miller D. (2003). Endophthalmitis caused by *Pseudomonas aeruginosa*. *Ophthalmology*.

[14] Obritsch M. D., Fish D. N., MacLaren R., Jung R. (2004). National surveillance of antimicrobial resistance in *Pseudomonas aeruginosa* isolates obtained from intensive care unit patients from 1993 to 2002. *Antimicrobial Agents and Chemotherapy*.

[15] Fuste E., Lopez-Jimenez L., Segura C., Gainza E., Vinuesa T., Vinas M. (2013). Carbapenem-resistance mechanisms of multidrug-resistant Pseudomonas aeruginosa. *Journal of Medical Microbiology*.

[16] Rodloff A. C., Goldstein E. J., Torres A. (2006). Two decades of imipenem therapy. *Journal of Antimicrobial Chemotherapy*.

[17] Freeman E. E. (2010). Rate of Endophthalmitis After Cataract Surgery in Quebec, Canada, 1996-2005. *JAMA Ophtalmology*.

[18] (2007). Prophylaxis of postoperative endophthalmitis following cataract surgery: results of the ESCRS multicenter study and identification of risk factors. *Journal of Cataract & Refractive Surgery*.

[19] Du D. (., Wagoner A., Barone S. B., Zinderman C. E., Kelman J. A., MaCurdy T. E., Forshee R. A., Worrall C. M., Izurieta H. S. (2014). Incidence of Endophthalmitis after Corneal Transplant or Cataract Surgery in a Medicare Population. *Ophthalmology*.

[20] Jabbarvand M., Hashemian H., Khodaparast M., Jouhari M., Tabatabaei A., Rezaei S. (2016). Endophthalmitis Occurring after Cataract Surgery. *Ophthalmology*.

[21] Aaberg T. (1998). Nosocomial acute-onset postoperative endophthalmitis survey A 10-year review of incidence and outcomes. *Ophthalmology*.

[22] Montan P. G., Koranyi G., Setterquist H. E., Stridh A., Philipson B. T., Wiklund K. (1998). Endophthalmitis after cataract surgery: risk factors relating to technique and events of the operation and patient history. *Ophthalmology*.

[23] Boks T. (2006). An outbreak of endophthalmitis after extracapsular cataract surgery probably caused by endotoxin contaminated distilled water used to dissolve acetylcholine. *British Journal of Ophthalmology*.

[24] Mateos I., Valencia R., Torres M. J., Cantos A., Conde M., Aznar J. (2006). Nosocomial Outbreak of
*Pseudomonas aeruginosa*
Endophthalmitis. *Infection Control & Hospital Epidemiology*.

[25] Arsan A. K., Adişen A., Duman S., Aslan B., Koçak, MD İ. (1996). Acute endophthalmitis outbreak after cataract surgery. *Journal of Cataract & Refractive Surgery*.

[26] Hoffmann KK., Weber DJ., Gergen MF., Rutala WA., Hill C., Tate G. (2002). Pseudomonas aeruginosaΓ, related postoperative endophthalmitis linked to a contaminated phacoemulsifier. Arch Ophthalmol. *Pseudomonas aeruginosaâ€*.

[27] Kenchappa P., Sangwan V., Ahmed N., Rao K. R., Pathengay A., Mathai A., Mansoori T., Das T., Hasnain S., Sharma S. *Annals of Clinical Microbiology and Antimicrobials*.

[28] Cruciani M., Malena M., Amalfitano G., Monti P., Bonom L. (1998). Molecular Epidemiology in a Cluster of Cases of Postoperative
*Pseudomonas aeruginosa*
Endophthalmitis. *Clinical Infectious Diseases*.

